# Épidémiologie du paludisme dans les armées françaises

**DOI:** 10.48327/mtsi.v3i1.2023.311

**Published:** 2023-02-21

**Authors:** Franck De Laval, Vincent Pommier De Santi

**Affiliations:** 1 Service de surveillance épidémiologique et investigations, Centre d’épidémiologie et de santé publique des Armées (CESPA), BdD Marseille-Aubagne 111, avenue de la Corse, BP 40026, 13568 Marseille Cedex 02, France; 2 Service de prévention du risque vectoriel, Centre d’épidémiologie et de santé publique des Armées (CESPA), BdD Marseille-Aubagne 111, avenue de la Corse, BP 40026, 13568 Marseille Cedex 02, France; * Actes du Colloque – Centenaire de la mort d'Alphonse Laveran. 24 novembre 2022, Paris / Proceedings of the Conference – Centenary of the death of Alphonse Laveran. 24 November 2022, Paris

**Keywords:** Alphonse Laveran, Forces armées, Opération, SSA, Service de santé des armées, Paludisme, *Plasmodium*, *Anopheles*, Moustique, Vecteur, Insecticide, Chimioprophylaxie, Côte d'Ivoire, Guyane, République centrafricaine, Alphonse Laveran, Armed Forces, Operation, SSA, French Defence Health Service, Malaria, *Plasmodium*, *Anopheles*, Mosquito, Vector, Insecticide, Chemoprophylaxis, Ivory Coast, French Guiana, Central African Republic

## Abstract

De par la morbidité et la létalité individuelles et l'incapacité collective qu'il peut produire, le paludisme a toujours représenté un risque pour les Forces armées en opération. La lutte contre le paludisme est un véritable plan de santé publique mené par le Service de santé des armées (SSA) au profit des Forces. Ce plan s'articule en quatre axes: la lutte antivectorielle qui vise les larves et moustiques adultes du genre *Anopheles*, la protection personnelle antivectorielle qui limite le contact homme-vecteur, la chimioprophylaxie, le diagnostic et le traitement précoce des paludismes.

Depuis 2001, l’épidémiologie du paludisme dans les armées a été rythmée par des épidémies de grande ampleur lors des engagements opérationnels en Côte d'Ivoire, en Guyane et en République centrafricaine. Le début d'une opération militaire s'accompagne d'enjeux stratégiques et logistiques prioritaires prenant le pas sur la prévention. En outre, l'application rigoureuse des mesures de protection individuelle reste difficile et encore plus en situation de combat.

Le développement du paludisme urbain en Afrique, l'utilisation d'une chimioprophylaxie causale, l'alternative au tout insecticide, et le développement d'outils diagnostiques performants permettant une prise en charge précoce et adaptée sont les défis à venir pour le SSA.

## Abstract (en)

Because of the individual morbidity and lethality and the resulting collective incapacity, malaria has always been a risk for the Armed Forces in operation. The fight against malaria is a real public health plan carried out by the Armed Forces Health Service (SSA) for the benefit of the Forces. This plan has four main components: vector control, which targets larvae and adult mosquitoes of the genus *Anopheles*, personal vector protection, which limits human-vector contact, chemoprophylaxis, and early diagnosis and treatment of malaria.

Since 2001, the epidemiology of malaria in the Armed Forces have suffered from large-scale epidemics during operational engagements in Côte d'Ivoire, Guyana and the Central African Republic. The start of a military operation is accompanied by strategic and logistical priorities that take precedence over prevention. In addition, the rigorous application of personal protection measures remains difficult and even more so in a combat situation.

The development of urban malaria in Africa, the use of causal chemoprophylaxis, the alternative to “nothing but insecticides”, and the development of efficient diagnostic tools allowing for early and adapted management are the challenges ahead for the SSA.

## Introduction

De par la morbidité et la létalité individuelles et l'incapacité collective qu'il peut produire, le paludisme a toujours été une menace pour les Forces armées en opération. Les situations où le paludisme a joué un rôle stratégique sont nombreuses dans l'Histoire. Pour les armées françaises on peut évoquer la campagne de Madagascar au xix^e^ siècle, ou la campagne de l'armée d'Orient pendant la Première Guerre mondiale. À cette dernière occasion les mesures mises en place par les frères Sergent, s'appuyant sur les résultats scientifiques d'Alphonse Laveran, posaient déjà les bases de la lutte contre le paludisme dans les armées [10]. Elles conservent aujourd'hui toute leur pertinence. Ainsi en ce début de xxi^e^ siècle, soit un siècle plus tard, les armées françaises sont toujours confrontées à cette menace principalement dans les déploiements en Guyane et en Afrique, et doivent mettre en place un véritable plan de santé publique mené par le Service de santé des armées (SSA) [11]. Ce plan s'articule en quatre axes principaux: la lutte antivectorielle (LAV) qui vise les larves et moustiques adultes du genre *Anopheles*, la protection personnelle antivectorielle (PPAV) qui limite le contact homme-vecteur, la chimioprophylaxie en prévention secondaire, et enfin le diagnostic et le traitement précoce des paludismes en prévention tertiaire des séquelles et des décès.

## La Lutte Contre Le Paludisme Dans Les Armées Au Xxi^e^ Siècle

La lutte contre le paludisme dans les armées se décline en quatre axes [8].

La LAV débute dès le choix de l'emplacement du bivouac ou du campement, idéalement situé à distance des réservoirs (les peuplements humains situés en zone d'endémie) et des zones propices au développement des vecteurs (les milieux humides). Pour des raisons opérationnelles ou logistiques, ces critères de choix ne peuvent pas toujours être respectés. L'entretien du bivouac est également fondamental: drainage, débroussaillage, gestion des déchets, et lutte physique contre les gîtes larvaires afin de limiter le développement et le repos des moustiques anophèles. En sus, est utilisé l’épandage de larvicides biologiques dans les gîtes non réductibles. L'utilisation des autres insecticides dans l'environnement, que ce soit en pulvérisation spatiale ou sur les surfaces, a longtemps été recommandée. Il ne s'agit actuellement plus d'une priorité du fait de leur toxicité et de la résistance acquise des vecteurs à ces produits, rendant le rapport bénéfice sur risque moins favorable.

La PPAV associe le port de vêtements couvrants pendant la période nocturne, en association avec l'utilisation de répulsif cutané sur la peau découverte. L'utilisation de la moustiquaire imprégnée d'insecticide à longue durée d'action est une autre mesure essentielle qui a fait preuve de son efficacité. La prise de la chimioprophylaxie pendant et autour du séjour en zone impaludée est aussi un axe majeur de la prévention du paludisme dans les armées. Elle permet de « combler les trous de la moustiquaire », mais elle est souvent la seule mesure réalisable en situation de mobilité et de combat, lorsque l'installation d'un bivouac n'est plus possible. La chimioprophylaxie est finalement « le dernier rempart » contre l'accès palustre. Actuellement, sont prescrits en première intention et quotidiennement la doxycycline ou l'atovaquone-proguanil, selon la durée d'exposition et la tolérance individuelle.

Du fait de difficultés logistiques à acheminer le matériel de protection, et/ou des comportements humains qui font fluctuer l'observance de la chimioprophylaxie, des accès palustres peuvent survenir chez les militaires. De plus, les espèces *Plasmodium vivax* et *P. ovale* ont des formes quiescentes hépatiques, pouvant entraîner des accès de reviviscence une fois la chimioprophylaxie antipaludique terminée. Il faut alors identifier ces accès précocement de manière à les prendre en charge avant toute aggravation.

Chacun a son rôle, le patient, le médecin et le laboratoire qui doivent chacun participer à réduire les délais, respectivement du recours aux soins, du diagnostic clinique, du diagnostic parasitologique et finalement de l'administration du traitement puis de la surveillance de l’évolution, en utilisant en première intention les traitements combinés à base d'artémisinine. Pour les paludismes à reviviscence, une cure radicale par primaquine est également requise.

En sus de ces quatre axes, le patient étant au centre de sa prévention et de sa prise en charge, des actions de promotion de la santé sont mises en place avant, pendant et après le séjour à risque.

La surveillance épidémiologique mesure de façon continue l'incidence et la situation du paludisme dans les armées. Elle permet d’évaluer l'efficacité du plan et d'orienter les priorités de recherche contre le paludisme dans les Forces [9].

## Le Paludisme Au Sein Des Forces ArmÉes En Guyane

La surveillance épidémiologique a permis d'identifier au sein des Forces armées en Guyane (FAG) une morbidité importante du paludisme, principalement à *P. vivax* [15], ce qui a poussé le SSA à mener un projet de recherche visant à améliorer la compréhension globale de l’épidémiologie du paludisme dans ce territoire. Le paludisme en Guyane était principalement une infection des zones d'orpaillage situées en forêt amazonienne [7, 12]. Les orpailleurs étaient les réservoirs; jusqu’à la moitié d'entre eux pouvaient être porteurs du parasite, majoritairement *P. falciparum*, dont 40% avec un portage asymptomatique de plasmodies [13]. Jusqu’à 10% des vecteurs y étaient infectés, et *Anopheles darlingi* avait un cycle d'agressivité s’étendant jusqu’à 15 heures sous le couvert de la canopée [14]. Les militaires et gendarmes, lorsqu'ils effectuent des opérations de lutte contre l'orpaillage illégal, investissent et occupent ces zones, et sont de fait exposés aux piqûres infectantes de nuit mais aussi de jour. À ce niveau de risque, les mesures PPAV ne peuvent prémunir totalement contre les piqûres infectantes et tout oubli de la chimioprophylaxie peut alors aboutir à un accès palustre.

Un programme de lutte innovant a permis de pallier l'accès aux soins des orpailleurs, difficile en forêt, en leur délivrant des tests de diagnostic rapide (TDR), des traitements antipaludiques et une moustiquaire imprégnée d'insecticide à longue durée d'action, ainsi qu'en les formant à leur utilisation [6]. Cette stratégie a permis d'agir directement sur le réservoir de parasite et s'est accompagnée d'une diminution du paludisme dans la population guyanaise et dans les FAG [16].

## Le Paludisme: Une Menace Pour Les Forces En Afrique

Le paludisme est endémique en Afrique, avec différents faciès épidémiologiques. Les Forces de présence françaises sont exposées de manière continue (au Gabon, en République centrafricaine, en Côte d'Ivoire) ou saisonnière (au Sahel, au Sénégal, à Djibouti).

En sus de ce fond de transmission endémique, les Forces françaises sont confrontées à une acmé du risque lors des ouvertures de théâtres d'opérations. Une ouverture de théâtre se produit toujours dans un pays en crise, avec souvent des populations déplacées et sans recours à la prévention et aux soins. Ces conditions sont propices au développement du paludisme. La projection des Forces avec toute la logistique attenante ne peut se faire immédiatement en totalité et le soutien logistique des unités combattantes arrive au fur et à mesure des possibilités. Cette conjonction aboutit à la projection de personnels naïfs avec une protection limitée dans un environnement où le niveau de transmission du paludisme est élevé. Là encore, tout oubli de la chimioprophylaxie peut aboutir à un accès palustre. Du fait de la fatigue et de l'intensité des missions, les militaires peuvent oublier la chimioprophylaxie et les épidémies survenir. Ce fut le cas lors de l'opération Licorne en Côte d'Ivoire ou de l'opération Sangaris en Centrafrique [4, 2].

Ces dernières années, la surveillance épidémiologique au sein des Forces françaises à Djibouti a identifié une augmentation progressive de l'incidence du paludisme, alors même que Djibouti était auparavant en phase de pré-élimination de la maladie. Un nouveau vecteur urbain, *An. stephensi*, a été identifié, importé d'Asie [5]. Il est résistant à toutes les familles d'insecticides et se développe dans des gîtes larvaires artificiels, ce qui lui permet de se maintenir pendant les conditions climatiques de la saison chaude. Cette émergence vectorielle dans la Corne de l'Afrique pourrait, si elle se propageait, modifier la transmission du paludisme dans les villes du continent, et avoir un impact majeur pour les populations et les Forces françaises présentes.

## Enjeux Et Perspective

D'une part, les vecteurs sont de plus en plus résistants aux insecticides et notamment aux pyréthrinoïdes de synthèse, seuls autorisés en Europe. D'autre part, la toxicité environnementale voire humaine de ces produits est maintenant décrite. En outre, la réglementation française impose une formation des personnels pour toute utilisation professionnelle des insecticides. Ceci complique leur utilisation en pulvérisation spatiale ou intradomiciliaire dans les Forces. De ce fait, la place des insecticides dans la lutte contre le paludisme dans les armées est en cours de réévaluation, hormis pour l'utilisation de la moustiquaire imprégnée d'insecticide à longue durée d'action qui reste une mesure efficace et préconisée. Cependant, il n'y a pas actuellement de mesures alternatives efficaces.

Ceci renforce le rôle de la chimioprophylaxie comme dernier rempart contre l'accès palustre. C'est aussi la mesure logistique la plus facile à mettre en place. L'amélioration de son observance et de son efficacité est un axe majeur d’évolution de la lutte. L'utilisation de la tafénoquine permettrait de passer à une prise hebdomadaire. Elle agit également sur les formes quiescentes des parasites à reviviscence. La tafénoquine est en cours d’évaluation au niveau de l'Agence européenne du médicament. Son utilisation dans les armées nécessiterait un dépistage préalable de l'activité enzymatique de la glucose-6-phosphate-déshydrogénase.

L'amélioration de la prise en charge constitue le dernier axe. L’épidémie de COVID-19 a en effet rappelé que l'allongement du délai jusqu'au traitement (ici du fait de la confusion entre accès palustre et COVID-19) entraînait une augmentation de la proportion des paludismes graves [3]. L’éducation pour la santé doit permettre de raccourcir le recours aux soins. La formation des soignants du SSA doit être renforcée pour qu'ils soient toujours au fait de la mise à jour des recommandations et en capacité de prendre en charge ces patients, tout particulièrement en situation isolée. La recherche et l'utilisation d'outils diagnostics plus performants doivent être une priorité. Par exemple, à Djibouti, l’émergence de souches de *P. falciparum* porteuses de délétions sur les gènes *pfhrp2/pfhrp3* diminue la sensibilité de certains TDR. L'utilisation de la biologie moléculaire sur le terrain, via le déploiement d'automates, pourrait améliorer la sensibilité du diagnostic [1]. Enfin la surveillance des résistances parasitaires aux différentes molécules utilisées permet d’être en mesure d'adapter les schémas prophylactiques ou thérapeutiques aux évolutions des parasites [17].

## Conclusion

L’épidémiologie du paludisme dans les armées est le résultat des projections des Forces dans des territoires aux faciès épidémiologiques différents (avec un niveau de transmission variable dans l'espace et dans le temps), et de l'application des mesures de prévention préconisées par le SSA. La lutte contre le paludisme dans les armées s'appuie sur une stratégie qui a fait preuve de son efficacité, mais qui doit s'adapter pour faire face aux nouvelles menaces: émergence de nouveaux vecteurs, résistance des vecteurs aux insecticides, multi-circulation de pathogènes, performance des outils diagnostics, résistances parasitaires.

Le SSA détient toutes les capacités nécessaires dans ces domaines, qu'il faudra maintenir si les Forces souhaitent conserver leur liberté de manœuvre en zone impaludée.

## Contribution Des Auteurs

FL et/ou VPS ont réalisé ou dirigé les différentes études dont les résultats ont été utilisés dans l'article.

FL et VPS ont conçu le projet de synthèse et écrit l'article.

## Liens D'intérêts

Les auteurs ne déclarent aucun conflit d'intérêts.

## References

[1] Bouzayene A, Zaffaroullah R, Bailly J, Ciceron L, Sarrasin V, Cojean S, Argy N, Houzé S, Joste V (2022). French National Malaria Reference Centre study group. Evaluation of two commercial kits and two laboratory-developed qPCR assays compared to LAMP for molecular diagnosis of malaria. Malar J.

[2] Créach MA, Velut G, de Laval F, Briolant S, Aigle L, Marimoutou C, Deparis X, Meynard JB, Pradines B, Simon F, Michel R, Mayet A (2016). Factors associated with malaria chemoprophylaxis compliance among French service members deployed in Central African Republic. Malar J.

[3] de Laval F, Maugey N, Bonet d'Oléon A, Pommier de Santi V, Ficko C (2021). Increased risk of severe malaria in travellers during the COVID-19 pandemic. J Travel Med.

[4] de Laval F, Simon F, Bogreau H, Rapp C, Wurtz N, Oliver M, Demaison X, Dia A, De Pina JJ, Merens A, Migliani R (2014). Emergence of Plasmodium ovale malaria among the French Armed Forces in the Republic of Ivory Coast: 20 years of clinical and biological experience. Clin Infect Dis.

[5] de Santi VP, Khaireh BA, Chiniard T, Pradines B, Taudon N, Larréché S, Mohamed AB, de Laval F, Berger F, Gala F, Mokrane M, Benoit N, Malan L, Abdi AA, Briolant S (2021). Role of Anopheles stephensi Mosquitoes in Malaria Outbreak, Djibouti, 2019. Emerg Infect Dis.

[6] Douine M, Sanna A, Galindo M, Musset L, Pommier de Santi V, Marchesini P, Magalhaes ED, Suarez-Mutis M, Hiwat H, Nacher M, Vreden S, Garancher L (2018). Malakit: an innovative pilot project to self-diagnose and self-treat malaria among illegal gold miners in the Guiana Shield. Malar J.

[7] Douine M, Sanna A, Hiwat H, Briolant S, Nacher M, Belleoud D, Le Tourneau FM, Bogreau H, De Laval F (2019). Investigation of a possible malaria epidemic in an illegal gold mine in French Guiana: an original approach in the remote Amazonian forest. Malar J.

[8] Experts du groupe de suivi permanent des questions relatives aux risques infectieux dans les armées Mémento Lutte contre le paludisme dans les armées. N° 514802/ARM/DCSSA/SDD/SES/NP du 14/12/2021.

[9] Michel R, Demoncheaux JP, Créach MA, Rapp C, Simon F, Haus-Cheymol R, Migliani R (2014). Prevention of infectious diseases during military deployments: a review of the French armed forces strategy. Travel Med Infect Dis.

[10] Migliani R, Meynard JB, Milleliri JM, Verret C, Rapp C (2014). Histoire de la lutte contre le paludisme dans l'armée française: de l'Algérie à l'Armée d'Orient pendant la Première Guerre mondiale. Med Sante Trop.

[11] Migliani R, Pradines B, Michel R, Aoun O, Dia A, Deparis X, Rapp C (2014). Malaria control strategies in French armed forces. Travel Med Infect Dis.

[12] Pommier de Santi V, Dia A, Adde A, Hyvert G, Galant J, Mazevet M, Nguyen C, Vezenegho SB, Dusfour I, Girod R, Briolant S (2016). Malaria in French Guiana Linked to Illegal Gold Mining. Emerg Infect Dis.

[13] Pommier de Santi V, Djossou F, Barthes N, Bogreau H, Hyvert G, Nguyen C, Pelleau S, Legrand E, Musset L, Nacher M, Briolant S (2016). Malaria Hyperendemicity and Risk for Artemisinin Resistance among Illegal Gold Miners, French Guiana. Emerg Infect Dis.

[14] Pommier de Santi V, Dusfour I, de Parseval E, Lespinet B, Nguyen C, Gaborit P, Carinci R, Hyvert G, Girod R, Briolant S (2017). Risque de transmission diurne du paludisme en forêt guyanaise. Med Sante Trop.

[15] Queyriaux B, Texier G, Ollivier L, Galoisy-Guibal L, Michel R, Meynard JB, Decam C, Verret C, Pommier de Santi V, Spiegel A, Boutin JP, Migliani R, Deparis X (2011). Plasmodium vivax Malaria among military personnel, French Guiana, 1998-2008. Emerg Infect Dis.

[16] Velut G, de Laval F, Delon F, d'Oléon A, Douine M, Mosnier E, Mmadi Mrenda B, Dia A, Musset L, Briolant S, Pommier de Santi V (2023). Sharp decrease in malaria incidence among the French armed forces in French Guiana. Travel Med Infect Dis.

[17] Wurtz N, Pascual A, Marin-Jauffre A, Bouchiba H, Benoit N, Desbordes M, Martelloni M, Pommier de Santi V, Richa G, Taudon N, Pradines B, Briolant S (2012). Early treatment failure during treatment of Plasmodium falciparum malaria with atovaquone-proguanil in the Republic of Ivory Coast. Malar J.

